# Reconstruction of a Plantar Defect Using a Split-Thickness Skin Graft: A Case Report and Review of Reconstructive Decision-Making

**DOI:** 10.7759/cureus.110617

**Published:** 2026-06-10

**Authors:** Ana Paula Rábago Jamaica, Erick R Guerra Bocanegra, José D Ortiz Cisneros, Juan C Martínez Nava, Fitzia M De Luna López, Farah M Hadad Monsiváis, Juan H Moreno Juárez, Ethan Villaseñor Cortina

**Affiliations:** 1 Surgery, Swiss Hospital, Monterrey, MEX

**Keywords:** acral melanoma, plantar reconstruction, sentinel lymph node, skin graft, split-thickness skin graft

## Abstract

Acral lentiginous melanoma presents reconstructive challenges due to its location in weight-bearing areas. We report the case of a 65-year-old female patient with plantar melanoma treated with wide local excision and sentinel lymph node biopsy. Reconstruction was performed using a split-thickness skin graft. The patient achieved complete graft integration without complications and preserved ambulation. This case supports that skin grafting should be considered a first-line reconstructive option in selected plantar defects, minimizing morbidity compared to flap-based techniques.

## Introduction

Acral lentiginous melanoma is a distinct subtype that frequently affects the plantar surface; it faces reconstructive challenges due to its biomechanical demands. Wide excision often results in defects requiring careful reconstructive planning [1,2]. 

Defects resulting from excision of plantar melanomas present significant reconstructive challenges because of the unique biomechanical properties of the plantar surface. The heel and plantar regions are constantly exposed to pressure, shear forces, and repetitive trauma during ambulation, while simultaneously possessing limited soft tissue mobility [1]. Reconstruction in these areas must therefore provide durable coverage capable of tolerating weight-bearing stress while preserving functional gait and minimizing morbidity [2]. 

Several reconstructive techniques have been described for plantar defects, including local flaps, regional flaps, free tissue transfer, and skin grafting. Flap-based reconstruction is often preferred for extensive or complex defects because it provides well-vascularized tissue with improved mechanical resistance. However, these procedures are associated with longer operative times, increased donor-site morbidity, and greater technical complexity [3]. In selected patients, split-thickness skin grafting may represent a simpler and less invasive alternative when an adequate vascularized wound bed is present. Recent reconstructive studies have demonstrated satisfactory functional and aesthetic outcomes using skin grafts in carefully selected foot and ankle defects [1]. 

In addition to local tumor control, accurate regional nodal staging is essential in melanoma management. Sentinel lymph node biopsy has become a standard component in the treatment of intermediate-thickness melanoma, providing important prognostic information and guiding further oncologic management. Large multicenter trials demonstrated the prognostic value and clinical utility of sentinel-node mapping compared with nodal observation alone [4,5].

We present the case of a patient with plantar acral lentiginous melanoma treated with wide local excision and sentinel lymph node biopsy, followed by reconstruction using split-thickness skin grafting with tie-over fixation, achieving satisfactory graft integration and preservation of ambulation.

## Case presentation

A 65-year-old female patient with a medical history significant for hypertension and type 2 diabetes mellitus presented with a pigmented lesion located on the plantar surface of the left foot.

Histopathological results confirmed acral lentiginous melanoma with a Breslow thickness of 2 mm and Clark level III. The lesion demonstrated close surgical margins (<1 mm), without evidence of ulceration or lymphovascular invasion.

Preoperative lymphoscintigraphy identified a sentinel lymph node in the left inguinal region. The patient subsequently underwent wide local excision according to oncologic guidelines, along with sentinel lymph node biopsy using a dual-tracer technique (radiotracer and patent blue dye) [3,4,5].

**Figure 1:**
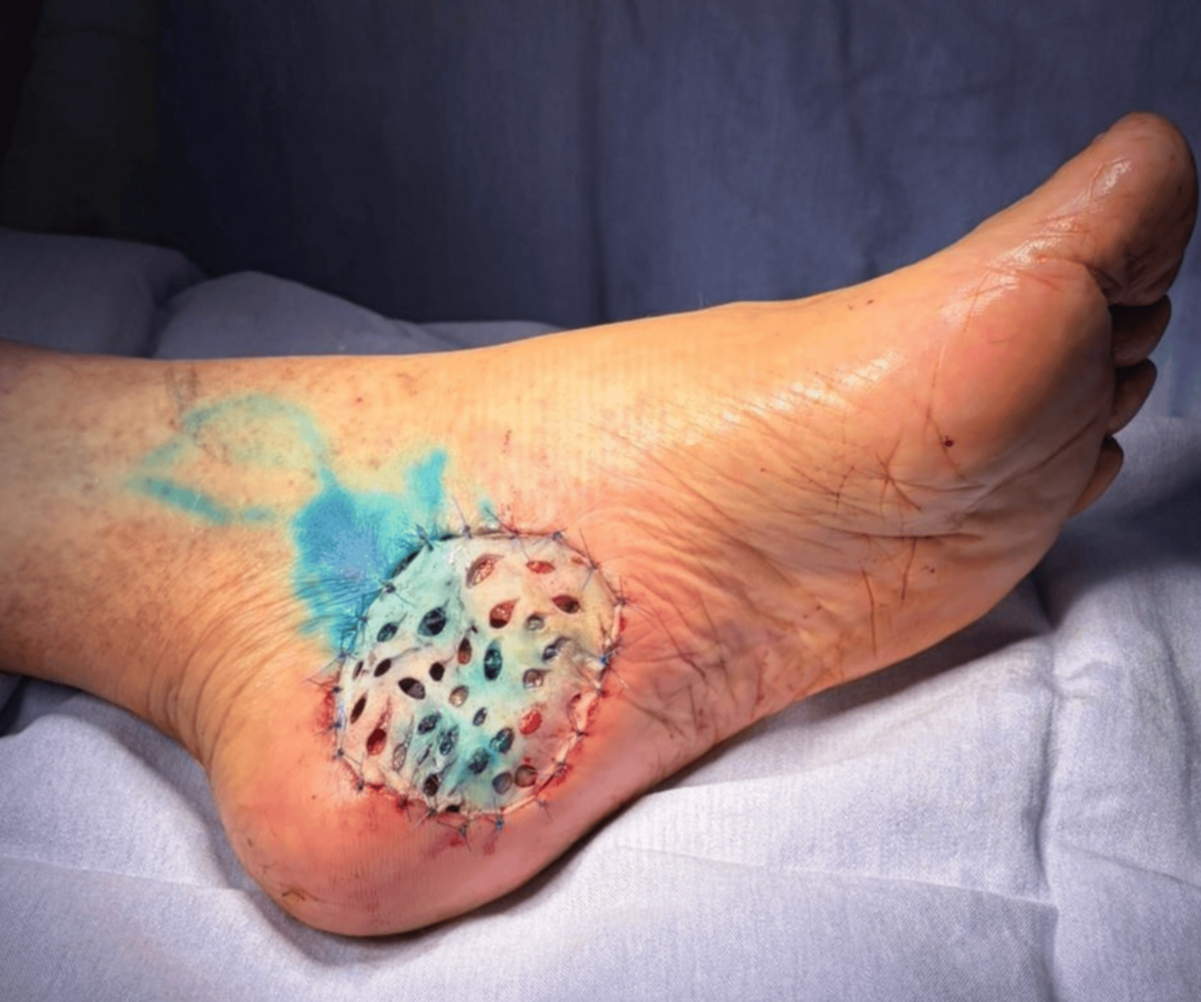
Application of the split-thickness skin graft over the plantar defect.

Following the tumor excision, a full-thickness soft tissue defect remained in the plantar calcaneal region. Given the characteristics of the defect, reconstruction was performed using a split-thickness skin graft (Figure 1).

The graft was secured in place and covered using the tie-over technique, consisting of non-adherent gauze impregnated with mupirocin ointment.

**Figure 2:**
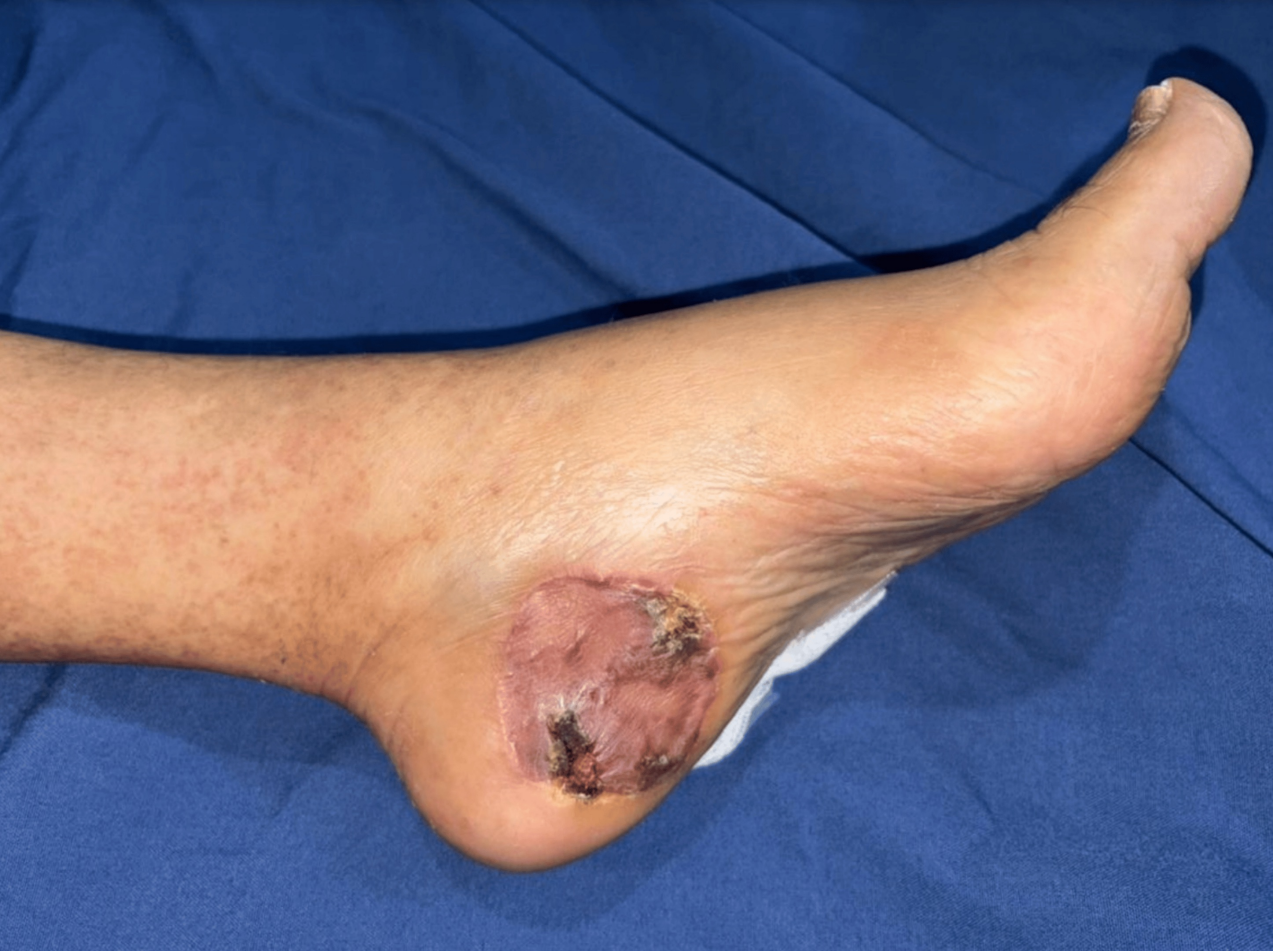
Skin graft integration progress on day 60.

The bolster dressing was maintained for seven days, after which it was removed. Upon evaluation 20, 30, and 60 days postoperatively (Figure 2), the graft demonstrated adequate integration, with appropriate adherence to the wound bed, no evidence of infection, necrosis, or hematoma, and satisfactory early take.

Therapeutic Intervention

The patient was placed in the supine position under epidural anesthesia. Local infiltration was performed around the melanoma site with appropriate oncologic margins on the left foot, followed by wide local excision of the lesion. Subsequently, left inguinal sentinel lymph node identification and excision were carried out using patent blue dye guidance, and the specimen was submitted for histopathological analysis.

A split-thickness skin graft was harvested from the right inguinal region. The graft was subsequently meshed and applied to the defect on the left foot. Fixation of the graft was achieved with interrupted 4-0 Prolene sutures (Ethicon, Somerville, NJ, USA). The graft was then covered with Italdermol™-impregnated gauze (Laboratori Baldacci S.p.A., Pisa, Italy) and secured using a tie-over dressing technique with interrupted 3-0 silk sutures. Adequate hemostasis was obtained throughout the procedure, with no intraoperative incidents or complications.

## Discussion

Reconstruction of plantar defects following melanoma excision represents a balance between oncologic safety and functional preservation. The plantar surface poses unique challenges due to its biomechanical demands, including constant pressure, shear stress, and limited soft tissue mobility. As a result, reconstruction must ensure both durable coverage and the ability to withstand weight-bearing forces [2].

Flap-based reconstruction, including local, regional, or free flaps, is often considered the standard for larger or complex defects due to its ability to provide vascularized and mechanically robust tissue. However, these techniques are associated with increased operative time, donor-site morbidity, and higher technical demands [3]. This is particularly relevant in patients with comorbidities such as diabetes mellitus, where minimizing surgical risk is essential.

Split-thickness skin grafting offers a simpler and less invasive alternative. Although traditionally considered less suitable for weight-bearing areas due to concerns regarding fragility and long-term durability, growing evidence supports its use in carefully selected plantar defects. Successful outcomes depend on several key factors, including adequate vascularization of the wound bed, meticulous surgical technique, and appropriate postoperative care [2].

In this case, favorable results can be attributed to proper patient selection and surgical planning. The defect, while located in a high-stress area, had a well-prepared vascularized base, allowing for successful graft take [1]. The use of a tie-over bolster dressing played a critical role by ensuring consistent pressure across the graft, reducing dead space, and preventing complications such as hematoma or seroma formation. Additionally, the use of mupirocin-impregnated gauze provided local antimicrobial coverage, which is particularly relevant in the plantar region.

However, the limitations of split-thickness skin grafts must be recognized. Grafts lack the thickness and mechanical resilience of native plantar skin, which may predispose to long-term breakdown under repetitive stress. Therefore, postoperative offloading, patient education, and close follow-up are essential to ensure durability and prevent recurrence of ulceration.

## Conclusions

Split-thickness skin grafting is a safe and effective reconstructive option for selected plantar defects following wide local excision of acral lentiginous melanoma. When combined with meticulous surgical technique and appropriate postoperative management, including tie-over fixation, a split-thickness skin graft can achieve reliable integration and preserve functional ambulation.

This approach should be considered as a first-line reconstructive strategy in carefully selected patients, offering reduced morbidity compared to flap-based techniques while maintaining satisfactory oncologic and functional outcomes.
